# Immune Profiling Enables Stratification of Patients With Active Tuberculosis Disease or *Mycobacterium tuberculosis* Infection

**DOI:** 10.1093/cid/ciaa1562

**Published:** 2020-10-16

**Authors:** Darragh Duffy, Elisa Nemes, Alba Llibre, Vincent Rouilly, Munyaradzi Musvosvi, Nikaïa Smith, Elizabeth Filander, Hadn Africa, Simbarashe Mabwe, Lungisa Jaxa, Bruno Charbit, Humphrey Mulenga, Michele Tameris, Gerhard Walzl, Stephanus Malherbe, Stephanie Thomas, Mark Hatherill, Nicole Bilek, Thomas J Scriba, Matthew L Albert, for the Milieu Intérieur Consortium, Laurent Abel, Andres Alcover, Hugues Aschard, Kalla Astrom, Philippe Bousso, Pierre Bruhns, Ana Cumano, Caroline Demangel, Ludovic Deriano, James Di Santo, Françoise Dromer, Gérard Eberl, Jost Enninga, Jacques Fellay, Odile Gelpi, Ivo Gomperts-Boneca, Milena Hasan, Serge Hercberg, Olivier Lantz, Claude Leclerc, Hugo Mouquet, Etienne Patin, Sandra Pellegrini, Stanislas Pol, Antonio Rausell, Lars Rogge, Anavaj Sakuntabhai, Olivier Schwartz, Benno Schwikowski, Spencer Shorte, Vassili Soumelis, Frédéric Tangy, Eric Tartour, Antoine Toubert, Mathilde Touvier, Marie-Noëlle Ungeheuer, Matthew L. Albert, Darragh Duffy, Lluis Quintana-Murci

**Affiliations:** 1 Immunobiology of Dendritic Cells, Institut Pasteur, Paris, France; 2 Inserm U1223, Institut Pasteur, Paris, France; 3 South African Tuberculosis Vaccine Initiative (SATVI), Division of Immunology, Department of Pathology and Institute of Infectious Disease and Molecular Medicine, University of Cape Town, Cape Town, South Africa; 4 DATACTIX, Paris, France; 5 Centre for Translational Research, Institut Pasteur, Paris, France; 6 Department of Science and Technology-National Research Foundation (DST-NRF) Centre of Excellence for Biomedical Tuberculosis Research, South African Medical Research Council Centre for Tuberculosis Research, Division of Molecular Biology and Human Genetics, Faculty of Medicine and Health Sciences, Stellenbosch University, Cape Town, South Africa; 7 Insitro, San Francisco, California, USA

**Keywords:** tuberculosis, immune profiling, patient stratification, cytokines, biomarkers

## Abstract

**Background:**

Tuberculosis (TB) is caused by *Mycobacterium tuberculosis* (*Mtb*) infection and is a major public health problem. Clinical challenges include the lack of a blood-based test for active disease. Current blood-based tests, such as QuantiFERON (QFT) do not distinguish active TB disease from asymptomatic *Mtb* infection.

**Methods:**

We hypothesized that TruCulture, an immunomonitoring method for whole-blood stimulation, could discriminate active disease from latent *Mtb* infection (LTBI). We stimulated whole blood from patients with active TB and compared with LTBI donors. *Mtb*-specific antigens and live bacillus Calmette-Guérin (BCG) were used as stimuli, with direct comparison to QFT. Protein analyses were performed using conventional and digital enzyme-linked immunosorbent assay (ELISA), as well as Luminex.

**Results:**

TruCulture showed discrimination of active TB cases from LTBI (*P* < .0001, AUC = .81) compared with QFT (*P* = .45, AUC = .56), based on an interferon γ (IFNγ) readout after *Mtb* antigen (Ag) stimulation. This result was replicated in an independent cohort (AUC = .89). In exploratory analyses, TB stratification could be further improved by the *Mtb* antigen to BCG IFNγ ratio (*P* < .0001, AUC = .91). Finally, the combination of digital ELISA and transcriptional analysis showed that LTBI donors with high IFNγ clustered with patients with TB, suggesting the possibility to identify subclinical disease.

**Conclusions:**

TruCulture offers a next-generation solution for whole-blood stimulation and immunomonitoring with the possibility to discriminate active and latent infection.

## Teaser

We tested TruCulture, an immunomonitoring tool, to identify active disease from latent *Mtb* infection. TruCulture showed improved discrimination of tuberculosis cases from LTBI as compared with QuantiFERON. Tuberculosis stratification could be further improved by the *Mtb* Ag:BCG IFNγ ratio.

Tuberculosis (TB) is a global public health problem, with an estimated 1.7 billion persons latently infected by *Mycobacterium tuberculosis* (*Mtb*) [1, 2]. Most newly infected individuals mount an effective immune response that controls infection; however, the host response does not fully eliminate the bacteria, resulting in a clinically asymptomatic state [3]. An estimated 5–10% of individuals with chronic infection progress to active disease at some point in their life, translating into approximately 10 million progressing to TB disease annually [3].

Due to the burden of infected persons in endemic regions and the high risk of reinfection, treatment strategies typically prioritize patients with active disease with the goal to limit transmission. As a result, there is a critical need to diagnose active disease and to distinguish it from latent infection. Diagnosis of active TB disease can be achieved by microscopy, polymerase chain reaction (PCR), or culture-based detection of *Mtb* presence in sputum. However, many patients with TB cannot produce sputum and it is preferable to utilize blood-based clinical assays, but available methods cannot reliably stratify active disease from latent infection. Whole-blood assays can distinguish infected from uninfected persons, based on stimulation with *Mtb* antigens (Ag), followed by an interferon γ (IFNγ) assay. Such assays include the QuantiFERON–TB Gold (QFT), which utilizes *Mtb*-specific antigens ESAT-6, CFP-10, and TB7.7 to stimulate immune cells in a blood collection tube with IFNγ secretion measured by enzyme-linked immunosorbent assay (ELISA), and T-SPOT.TB, which uses similar antigens with IFNγ measured by ELISPOT. Potential sources of technical variability with QFT may include the range of blood volumes (0.8–1.2 mL) and incubation times (16–24 hours) permitted in the manufacturer’s protocol, and recent studies have addressed these sources of variance [4, 5]. Despite these improvements, QFT has limitations for use as a diagnostic in TB-endemic countries, although it has been recently used to measure the efficacy endpoint in a prevention of *Mtb* infection phase II vaccine trial [6] and is used to assess inclusion/exclusion criteria [7, 8].

We have previously described use of TruCulture (TruC) devices, a syringe-based whole-blood collection and incubation system that allows immunomonitoring in response to diverse immune agonists, using proteomic [9] or transcriptional [10, 11] assays. Specifically, we demonstrated greater reproducibility in multicenter studies, as compared with peripheral blood mononuclear cell stimulation [12]. Given these findings in healthy donors, we evaluated whether TruC is applicable for immunomonitoring of patients with TB. As shown herein, we demonstrated the ability to more accurately classify patients with active disease and latently infected persons using TruC.

## METHODS

### Participant Groups

Twenty-five healthy adults with asymptomatic, latent *Mtb* infection (LTBI), defined by a positive QFT In-Tube (QFT+) assay (Qiagen, Germany), and 25 adults without human immunodeficiency virus (HIV) with TB disease, defined by a positive sputum XpertMTB/RIF test (Cepheid, USA) were identified and recruited at the South African Tuberculosis Vaccine Initiative (SATVI), Worcester, South Africa [13]. For the replication cohort, patients with TB (n = 51) enrolled in the PREDICT trial (clinicaltrials.gov NCT02821832) at SATVI were co-enrolled into this biomarker study. The LTBI controls (n = 9), recruited at SATVI, and healthy donors (n = 10), recruited in Paris, France, were also included. The TB clinical studies, protocols, and informed-consent forms were approved by the Human Research Ethics Committee of the University of Cape Town (reference 234/2015). Written informed consent was obtained from all study participants. Additional cohort details are provided in the Supplementary Methods and Table 1.

**Table 1.** Patient Characteristics

Initial Cohort	Patients With TB	LTBI Controls	*P*
Age, median (IQR), y	33 (25–40)	33 (24–41)	.89
Sex, % female	24	24	>.99
Ethnicity, % Cape mixed ancestry^a^	72	76	>.99
Household TB contact, % yes	56	40	.26
BMI, median (IQR), kg/m^2^	20 (19–21)	26 (24–28)	<.0001
Smoking status, n			
** **Smoker	14	13	.07
** **Ex-smoker	7	2	
** **Nonsmoker	4	10	
No. of donors			
** **Visit 1	25	25	NA
** **Visit 2	18 (after treatment)	19	NA

Abbreviations: BMI, body mass index; IQR, interquartile range; LTBI, latent *Mycobacterium tuberculosis* infection; NA, not applicable; TB, tuberculosis.

^a^Referred to as “coloured” in South Africa.

### Whole-blood Stimulations

TruC tubes (Myriad RBM) were batch-prepared and maintained at −20°C until time of use. To prepare TruC TB antigen tubes, 3 QFT TB antigen tubes (the QFT Gold In-tube system was used, as the study was performed prior to the introduction of the QFT Gold Plus) were rinsed with 2 mL of TruC media and the media transferred into empty TruC tubes to maintain the same concentration of *Mtb* antigens as found in QFT. Live bacillus Calmette-Guerin (BCG; Connaught strain, Sanofi Pasteur) tubes were prepared to have a final concentration of 10^5^ bacteria/mL.

### Multianalyte Protein Profiling

Supernatants from QFT and TruC tubes were analyzed for IFNγ by standard ELISA (Qiagen) and values were expressed in IU/mL, calculated by subtraction of values from the relevant nonstimulated controls and normalized for the dilution factor. Luminex xMAP technology was used to measure 32 proteins in the same samples (Myriad RBM). To detect low concentrations of IFNγ, a homebrew Simoa ELISA was developed as previously described [14] and detailed in the Supplementary Methods.

### Nanostring Transcriptional Analysis

Nanostring gene expression analysis (Human Immunology V2 panel plus 30 TB-related genes listed in Supplementary Table 1) was performed following extraction of RNA from Trizol-stabilized TruC cell pellets as previously described and detailed in the Supplementary Methods [10].

## RESULTS

### Improved Discrimination of Patients Using TruC Tuberculosis Antigen (Ag) Stimulation

**Figure 1.**
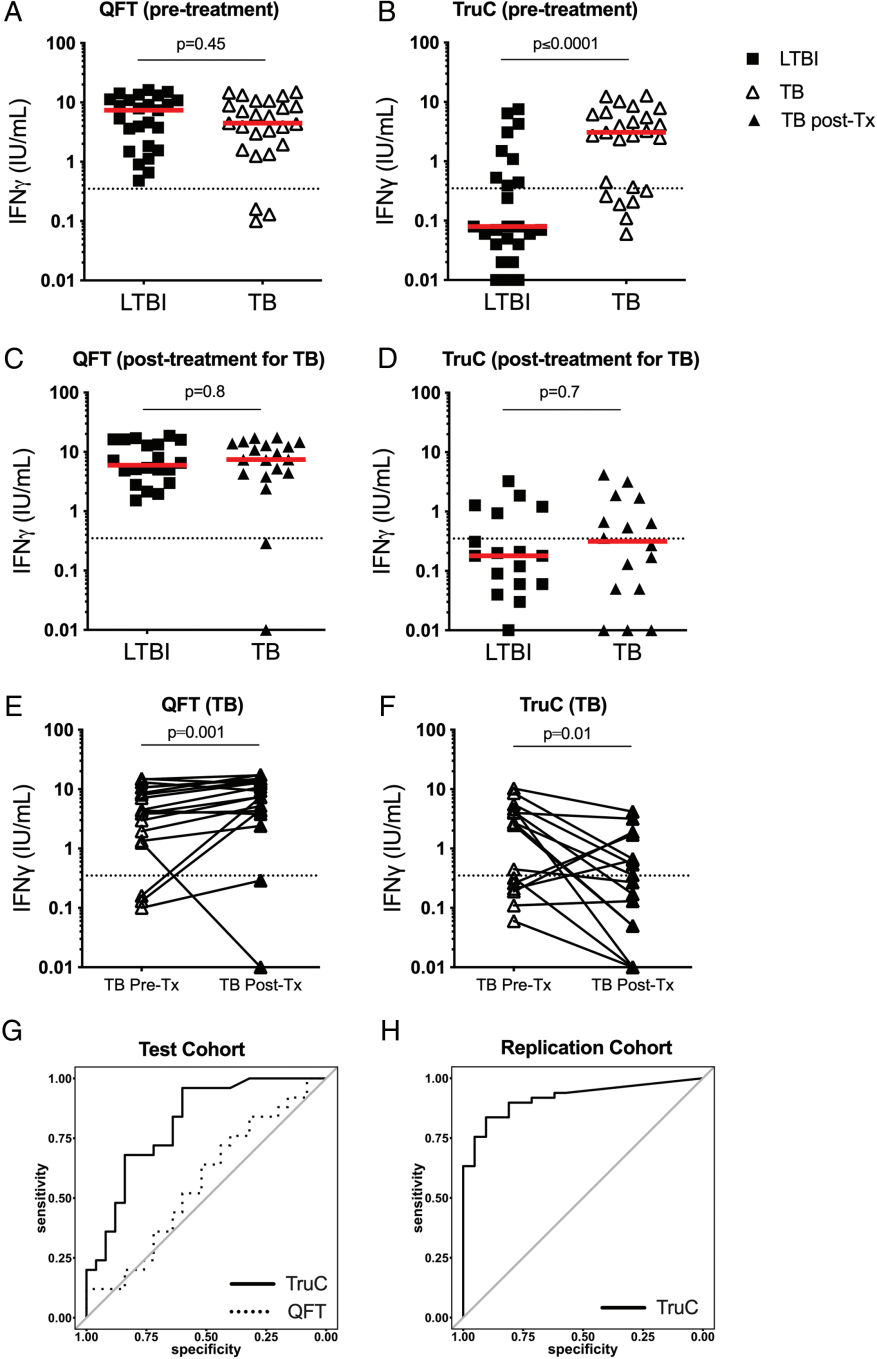
IFNγ *Mtb* Ag response. IFNγ response following *Mtb* Ag stimulation and subtraction of the null control in patients with LTBI and TB in QFT tubes pretreatment (*A*), TruC tubes pretreatment (*B*), QFT tubes after successful antibiotic treatment in patients with TB (*C*), and TruC tubes after successful antibiotic treatment in patients with TB (*D*). *a*, *b*: n = 25/25; *c*, *d*: n = 19/18 LTBI/TB, Mann Whitney; bars represent the median values, the dotted line is the QFT positive cutoff at 0.35 IU/mL. Paired IFNγ responses following *Mtb* Ag stimulation and subtraction of the null control in patients with TB pre- and posttreatment in QFT tubes (*E*) and TruC tubes (*F*) (paired *t* test). *G*, ROC curve analysis of the IFNγ response to classify active disease following *Mtb* Ag stimulation in TruC (black lines) or QFT (dashed lines) tubes in the initial cohort; *H*, ROC curve analysis of the IFNγ response to classify active disease following *Mtb* Ag stimulation in TruC in a blinded independent replication study (n = 80). TB: black squares; LTBI: open triangles; TB post-treatment: black triangles. Abbreviations: Ag, antigen; IFNγ, interferon γ; LTBI, latent *Mycobacterium tuberculosis* infection; *Mtb*, *Mycobacterium tuberculosis*; post-Tx, post-treatment; QFT, QuantiFERON; ROC, receiver operating characteristic; TB, tuberculosis; TruC, TruCulture.

To enable comparison between TruC and QFT, we transferred *Mtb* antigens from QFT into TruC tubes as described (see Methods). We sampled blood from patients with active TB and persons with LTBI, and measured induced IFNγ production utilizing ELISA. Confirming previous reports [15], QFT assays did not stratify TB and LTBI groups (Figure 1A) (*P* = .45). In contrast, TruC using the same *Mtb* antigens and IFNγ readout showed a significantly higher response in patients with TB as compared with LTBI controls (*P* < .0001) (Figure 1B). Inclusion criteria for defining LTBI cases were based on historical QFT positivity (IFNγ > 0.35 IU/mL), confirmed upon re-testing (Figure 1A). Indicating distinct parameterization between the 2 assays, when this predefined cutoff was applied to the TruC results, only 9 LTBI cases and 17 patients with TB scored positive (Figure 1B).

All patients with active disease were treated and 18 agreed to retesting 12–18 months later, all of whom had a successful treatment outcome. We also retested 19 LTBI controls after a similar 12–18-month time interval. At this time point no differences were observed between the LTBI and treated patients with TB with either QFT or TruC systems (Figure 1C and 1D). When the effect of treatment on patients with TB was directly examined, both QFT and TruC assays showed significant differences (pre- vs post-treatment, paired *t* test) (Figure 1E and 1F). Paradoxically, patients showed an increased IFNγ response in QFT (*P* = .001) when comparing post versus pretreatment cytokine levels, whereas the majority of patients showed the expected decrease in IFNγ responses as measured by TruC (*P* = .01). TruC pretreatment results had an area under the receiver operating characteristic (ROC) curve (AUC) of .814 (95% confidence interval [CI], .69–.93), in comparison to .563 (95% CI, .40–.72) for QFT (Figure 1G). A bootstrap test between the ROC curves showed a statistically significant improvement for TruC compared with QFT (*P* = .04). To replicate the TruC result, we recruited an independent cohort of actively infected patients with TB (n = 51), healthy LTBI controls (n = 9), and healthy nonendemic donors (n = 10) to test the ability of TruC *Mtb* Ag stimulation to correctly classify active disease. In this blinded study TruC *Mtb* Ag induced IFNγ had an AUC = .89 (95% CI, .82–.97) for identification of patients with TB disease (Figure 1H).

### Multiple Cytokine Responses Stratify Active Tuberculosis and Latent Infection After TruC *Mtb* Ag Stimulation

**Figure 2.**
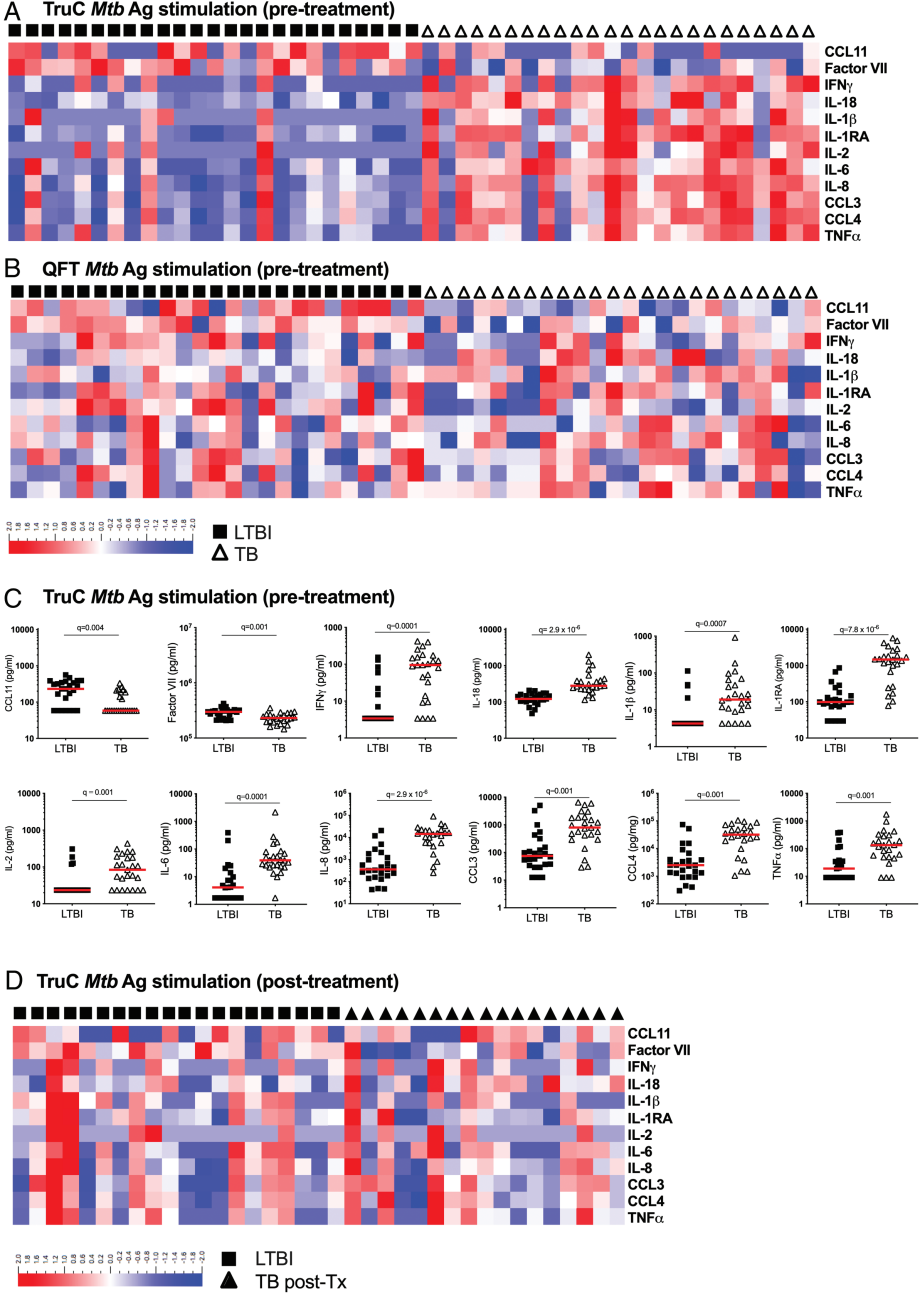
Differential cytokine responses in *Mtb* infection versus TB disease. Heatmaps of relative expression levels for 12 differential cytokines (LTBI vs TB groups, Mann-Whitney *q* < 0.01) segregated by patient group (LTBI: black squares; TB: open triangles) after *Mtb* Ag stimulation in TruC (*A*) or QFT (*B*) tubes prior to treatment, and (*D*) TruC *Mtb* Ag stimulation after successful antibiotic treatment of the TB patient group (TB post-treatment: closed triangles). *C*, Dot plot representations of the differential cytokine concentrations between LTBI and TB groups (*q* < 0.01) in TruC tubes prior to treatment. *a*, *b*, *c*: n = 25/25; *e*: n = 19/18 latent/active; bars represent the median values, *q* value: FDR-corrected Mann-Whitney test). Abbreviations: Ag, antigen; FDR, false discovery rate; IFNγ, interferon γ; IL, interleukin; LTBI, latent *Mycobacterium tuberculosis* infection; *Mtb*, *Mycobacterium tuberculosis*; post-Tx, post-treatment; QFT, QuantiFERON; TB, tuberculosis; TNFɑ, tumor necrosis factor ɑ; TruC, TruCulture.

To assess the value of measuring additional inflammatory cytokines, we performed Luminex multi-analyte profiling on all supernatants. This identified 12 proteins that were differentially (*q* < 0.01) expressed between TB and LTBI groups (Figure 2A and 2C, Supplementary Table 2) in the *Mtb* antigen TruC supernatants, whereas only interleukin (IL) 2 was different in the respective QFT assays with this stringent cutoff (Figure 2B, Supplementary Table 2). A heatmap representation of the TruC results illustrates 10 proteins with higher responses (IFNγ, IL-18, IL-1RA, IL-2, IL-6, IL-8, chemokine (C-C motif) ligand [CCL3], CCL4, tumor necrosis factor α [TNFα]) and 2 with lower responses (CCL11, factor VII) in active TB as compared with patients with LTBI (Figure 2A), with no discernible pattern observed in the QFT stimulations for these cytokines (Figure 2B). Individual plots of protein concentrations are depicted for differential cytokines observed with TruC (Figure 2C) and QFT (Supplementary Figure 1). Following successful treatment of the TB group there were no significant differences between treated patients with TB and LTBI controls (Figure 2D). This analysis indicated that TruC stimulation could reveal multiple immune perturbations in active TB disease.

### QFT Negative-control Tubes Have High Nonspecific Cytokine Activation

**Figure 3.**
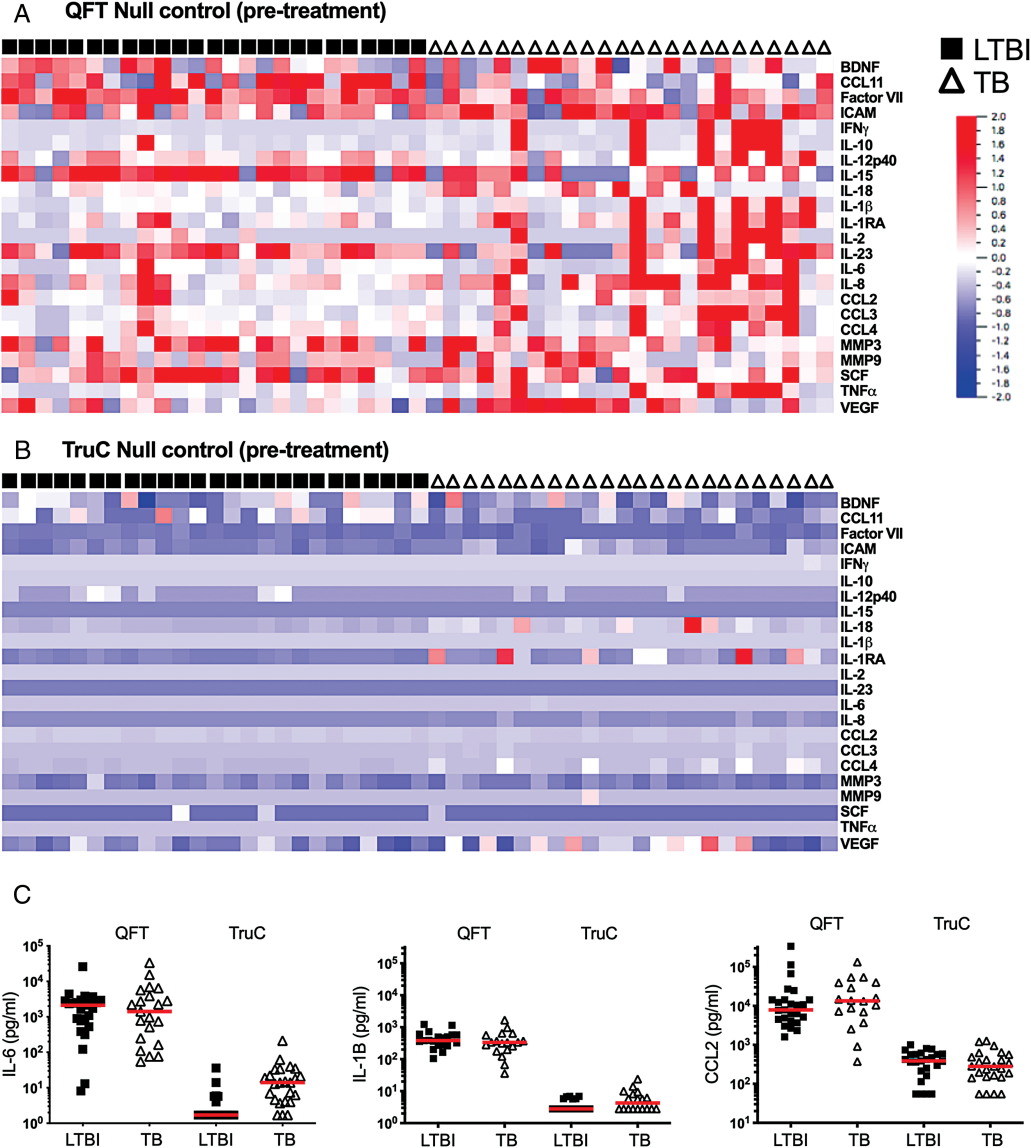
Differential cytokines in QFT and TruC null tubes. Heatmaps of relative cytokine expression levels segregated by patient group (LTBI: black squares; TB: open triangles) in QFT (*A*) and TruC null tubes (*B*) for 22 out of 32 cytokines measured, selected based on variance (σ/σ _max_ = 0.138). *C*, Concentrations of IL-6, IL-1β, and CCL2 in QFT and TruC null tubes in patients with LTBI and TB. n = 25/25; bars represent the median values_._ Abbreviations: BDNF, brain derived neurotrophic factor; ICAM, intercellular adhesion molecule IFNγ, interferon γ; IL, interleukin; LTBI, latent *Mycobacterium tuberculosis* infection; MMP, matrix metallopeptidase; QFT, QuantiFERON; TB, tuberculosis; SCF, stem cell factor; TNFɑ, tumor necrosis factor ɑ; TruC, TruCulture; VEGF, vascular endothelial growth factor.

To examine underlying differences between TruC and QFT, we considered the nonspecific activation using the null control conditions. To avoid potential artefacts caused by outlier measurements, we performed prefiltering based on variance (σ/σ _max _= 3.25 × 10^−5^), which led to the removal of 9 proteins that showed low variance across all conditions. Analysis of the remaining 23 proteins revealed significant differences (null conditions QFT vs TruC, *q* < 0.01) with all proteins showing higher concentrations in the QFT tubes (Figure 3A and 3B, Supplementary Tables 3 and 4). IL-6, IL-1β, and CCL2 were the 3 most differentially expressed proteins (Figure 3C). Notably, these differences were independent of disease status, as all proteins remained significantly different after regressing for patient status (TB or LTBI).

**Figure 4.**
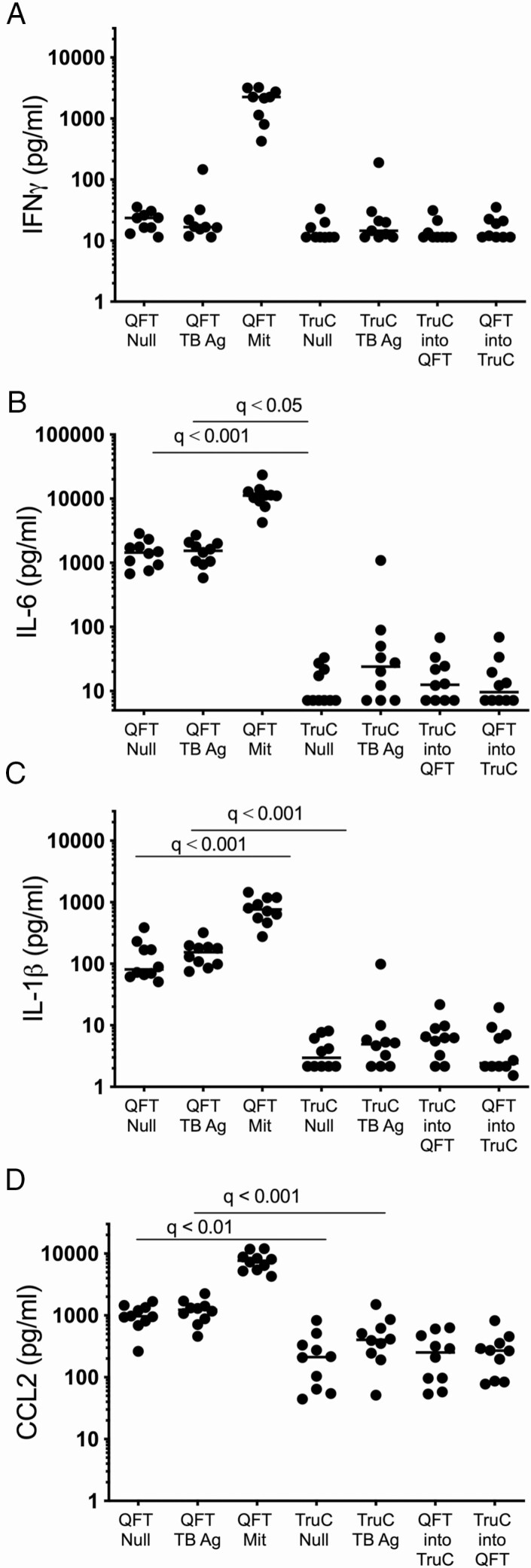
Cytokine responses in donors from a nonendemic TB region. Concentrations of IFNγ (*A*), IL-6 (*B*), IL-1β (*C*), and CCL2 (*D*) in QFT null, QFT TB Ag, QFT Mit, TruC null, TruC TB Ag, TruC BCG, and mixed cultures of TruC-QFT and QFT-TruC null conditions in healthy donors from a nonendemic region (n = 10; bars represent the median values, Friedman test with Dunn’s multiple-comparison test). Abbreviations: Ag, antigen; BCG, bacillus Calmette-Guérin; IFNγ, interferon γ; IL, interleukin; Mit, Mitogen; QFT, QuantiFERON; TB, tuberculosis; TruC, TruCulture.

To further validate and provide interpretability for this analysis, we performed additional experiments in healthy, *Mtb*-uninfected European donors. The same QFT and TruC stimulations were performed as described above. Additionally, we investigated the hypothesis that the QFT tube or the TruC media might account for the observed variability between the null conditions. We tested conditions in which blood collection was performed in the TruC tubes followed by transfer of the blood/media mixture into a QFT null tube, as well as the converse—blood collection and mixing in QFT tubes followed by transfer into a TruC null tube containing media in the absence of stimuli. A comparison between QFT and TruC negative-control tubes and *Mtb* Ag tubes showed results similar to the patients with TB and LTBI controls, with significantly higher levels of innate cytokines in QFT (IL-6, IL-1β, and CCL2 shown for comparison with prior results in Figure 3). Strikingly, in both of the tube transfer conditions, cytokine levels reflected the TruC condition and indicated that the TruC media minimized nonspecific innate cell activation observed when using QFT tubes. Unexpectedly, 1 donor showed elevated IFNγ responses in both stimulation systems (Figure 4A); however, the fold-change of the *Mtb* Ag over the null response was 4-fold in QFT and 16-fold in TruC, illustrating the improved signal-to-noise achievable for induced antigen-specific immune responses using TruC. This particular donor was also an outlier for other cytokine responses (eg, IL-6, IL-1β, CCL2) in *Mtb* Ag TruC stimulations (Figure 4B–D), but further clinical and radiological investigations ruled out TB disease. These combined results removed possible confounding factors due to *Mtb* infection and demonstrated that TruC media and the conditions reported facilitate an improved method for immune stimulation and immune monitoring.

### TruC Bacillus Calmette-Guérin Stimulation Revealed Additional Immune Response Differences and Improved Patient Classification

**Figure 5.**
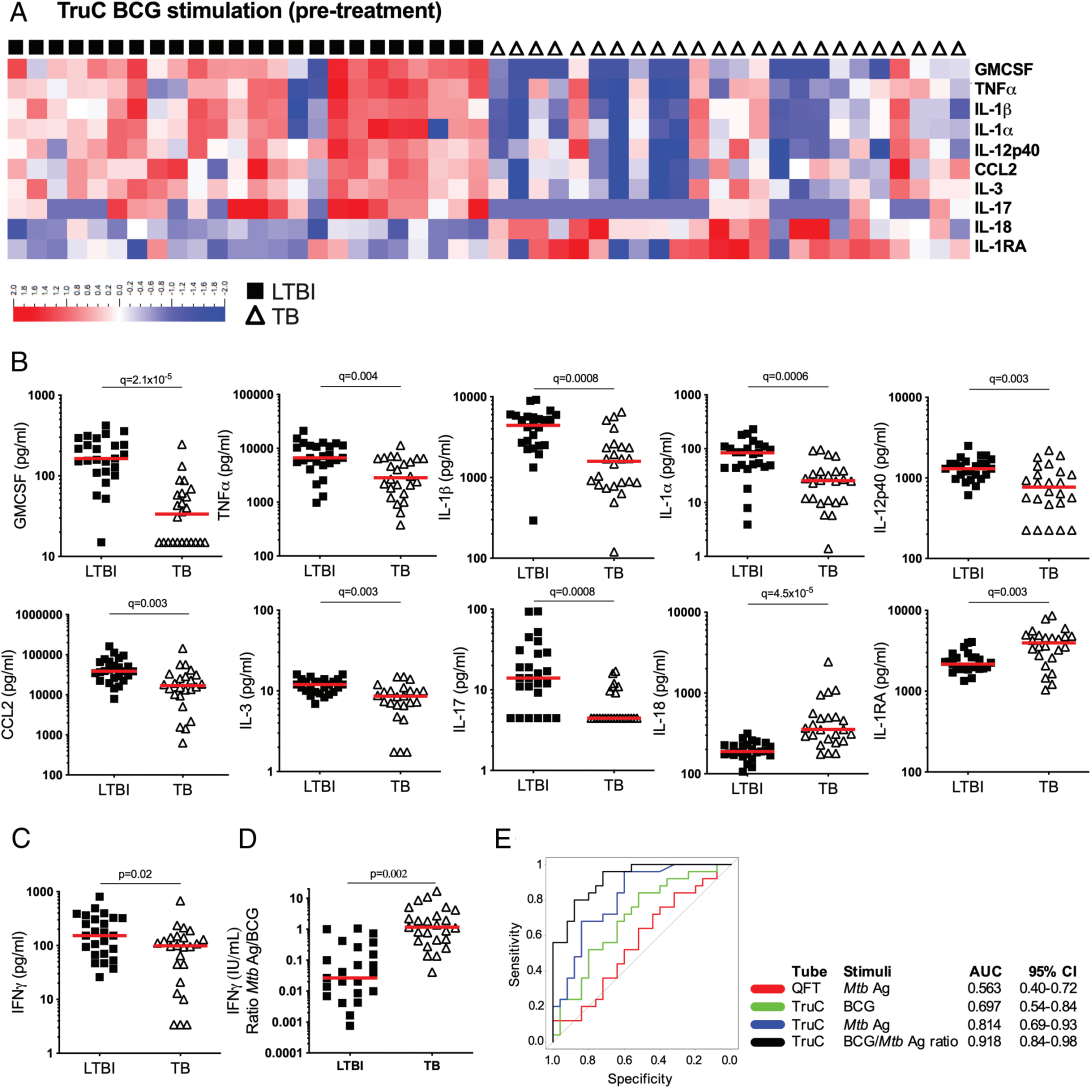
BCG-induced immune responses in *Mtb* infection versus TB disease. *A*, Heatmap of relative cytokine expression levels segregated by patient group (LTBI: black squares; TB: open triangles) after BCG TruC stimulation, and identification of differential proteins after FDR-adjusted Mann-Whitney tests between LTBI and TB groups. *B*, Dot plot representations of the cytokine concentrations of differential proteins between LTBI and TB groups: GMCSF, TNFα, IL-1β, IL-1α, IL-12p40, CCL2, IL-3, IL-17, IL-18, IL-1RA. (*C*) IFNγ BCG response and (*D*) ratio (IU/mL) of *Mtb* Ag/BCG stimulation for patients with LTBI and TB. *E*, ROC curve analysis of IFNγ ratio to *Mtb* Ag/BCG stimulation (black), IFNγ concentrations of TruC *Mtb* Ag (blue), TruC BCG (green), and QFT *Mtb* Ag (red) stimulations. n = 25/25; bars represent the median values; *q* value: FDR-corrected Student’s *t* test). Abbreviations: Ag, antigen; AUC, area under the receiver operating characteristic curve; BCG, bacillus Calmette-Guérin; CI, confidence interval; CCL, chemokine (C-C motif) ligand; FDR, false discovery rate; GMCSF, granulocyte-macrophage colony-stimulating factor; IFNγ, interferon γ; IL, interleukin; LTBI, latent *Mycobacterium tuberculosis* infection; *Mtb*, *Mycobacterium tuberculosis*; QFT, QuantiFERON; ROC, receiver operating characteristic; TB, tuberculosis; TNFɑ, tumor necrosis factor ɑ; TruC, TruCulture.

Given its use as a TB vaccine and its ability to trigger an innate response in whole blood [16], we explored the use of BCG as an additional TruC stimulation condition in our comparison study of TB and LTBI groups. Of the 22 proteins that were induced by BCG, 10 were differentially expressed (*q* < 0.01) between the 2 groups (Figure 5A and 5B, Supplementary Table 5). In contrast to TB Ag responses, the BCG-induced differences were mostly higher in the LTBI group (except for IL-18 and IL-1RA), with 4 of the most differentially expressed proteins being IL-1 family members (IL-1α, IL-1β, IL-1RA, and IL-18) (Figure 5B). Interestingly, IFNγ was only nominally higher in the LTBI group (*P* = .02) (Figure 5C). Again, the immune responses in patients with TB normalized after treatment compared with those seen in LTBI controls (Supplementary Figure 2).

Given that the pattern of BCG stimulation was inverse to that observed using *Mtb* antigen (ie, higher in LTBI compared with TB), we predicted that BCG-induced cytokines could be leveraged for improving the stratification of patient groups. We therefore calculated a composite index, the ratio of *Mtb* Ag and BCG-induced IFNγ response, which showed a more than 10-fold difference between the 2 patient groups (*P* = .002) (Figure 5D) and an AUC of .918 (95% CI, .84–.98) (Figure 5E). Notably, this AUC was superior to those achieved for the individual tests: TruC *Mtb* Ag (AUC, .814; 95% CI, .69–.93) or BCG (AUC, .697; 95% CI, .54–.84) and the QFT *Mtb* Ag (AUC, .563; 95% CI, .40–.72); and a bootstrap test for correlated ROC curves revealed statistically significant improvements over both TruC *Mtb* Ag (*P* = .02) and QFT *Mtb* Ag (*P* < .0001). These findings demonstrate the potential advantage of combining peptide antigen and complex stimuli for improved patient classification.

### Differential IFNγ Responses to Tuberculosis Ag and Bacillus Calmette-Guérin Stimulation

**Figure 6.**
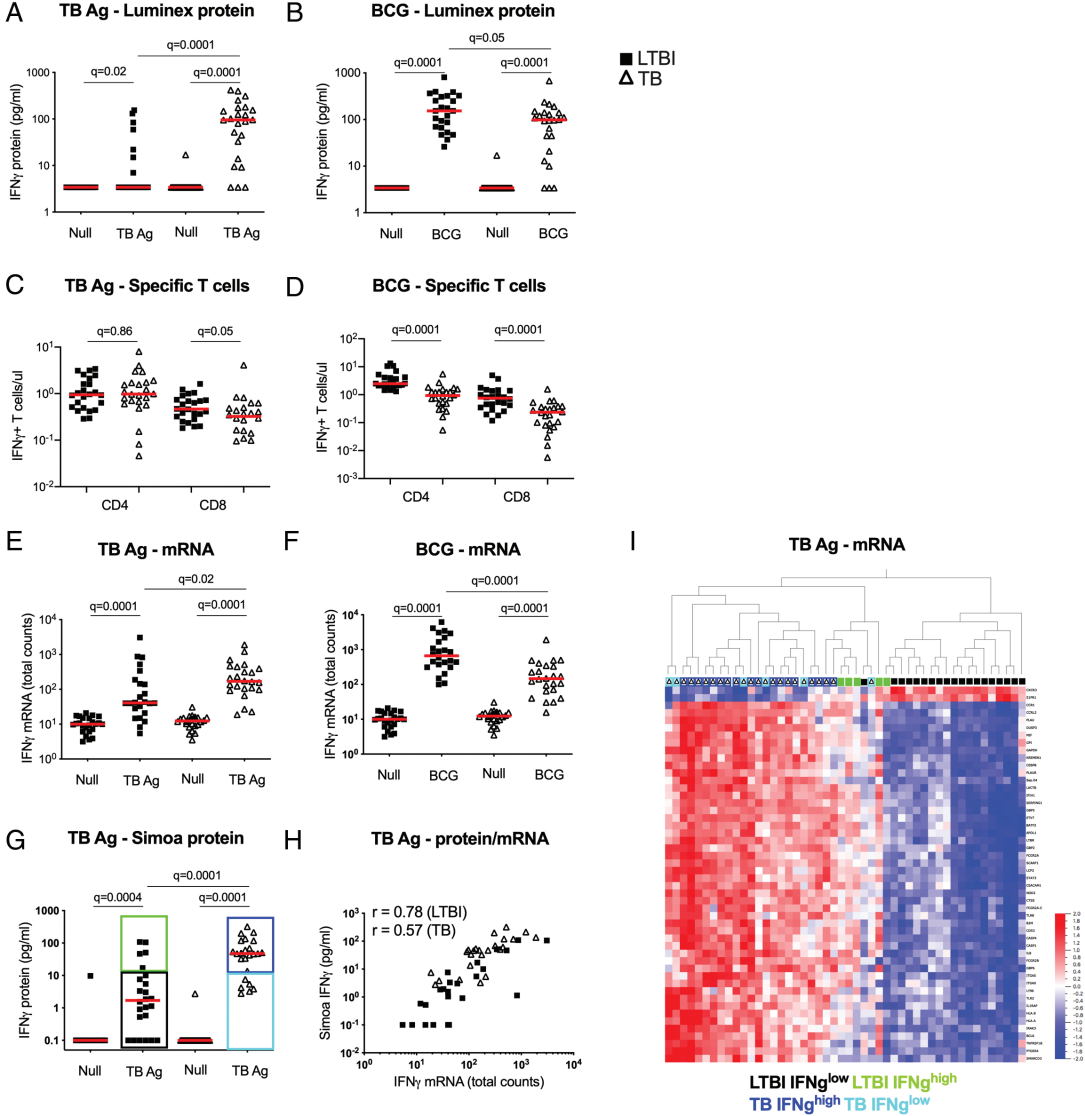
Differential IFNγ responses to TB Ag and BCG stimulations. IFNγ protein levels measured by Luminex in TruCulture supernatants after TB Ag (*A*) and BCG (*B*) stimulations. Total numbers of IFNγ+ CD4+ and IFNγ+ CD8+ T cells measured by flow cytometry in TB Ag (*C*) and BCG-stimulated whole blood (*D*). IFNγ mRNA levels measured by nanostring in TruCulture cell pellets after TB Ag (*E*) and BCG (*F*) stimulations. *G*, IFNγ levels measured by Simoa digital ELISA in TruCulture supernatants after TB Ag stimulation. Classification in 4 different groups according to disease status and IFNγ levels: LTBI IFNγ ^low^ (black), LTBI IFNγ ^high^ (green), TB IFNγ ^high^ (dark blue) and TB IFN^low^ (light blue). *H*, Correlation plot between IFNγ protein levels measured by Simoa and IFNγ mRNA total counts measured by nanostring, after TB Ag stimulation (Pearson correlation). *I*, Heatmap showing the 50 most differentially expressed genes between TB and LTBI for the TB Ag stimulation (unsupervised hierarchical clustering). Individuals are coded according to disease status and levels of IFNγ secretion, as illustrated in panel *G*. Solid lines depict medians. Comparisons of LTBI/TB groups within the same stimulation were performed using unpaired Mann-Whitney tests; comparisons between null and stimulated conditions within the LTBI/TB groups were performed using a Wilcoxon test. Correction for multiple comparisons was then applied. LTBI: black squares; TB: open triangles. Abbreviations: Ag, antigen; BCG, bacillus Calmette-Guérin; ELISA, enzyme-linked immunosorbent assay; IFNγ, interferon γ; LTBI, latent *Mycobacterium tuberculosis* infection; TB, tuberculosis.

Finally, to investigate further the differential responses observed between TB Ag and BCG-induced IFNγ (Figure 6A and 6B) we tested whether this reflected differing numbers of circulating antigen-specific T cells. For this, we examined previously published intracellular cytokine flow cytometry data from the same donors [13]. No significant differences in the total numbers (Figure 6C and 6D) or frequencies (Supplementary Figure 3*A*) of IFNγ ^+^ CD4^+^ and CD8^+^ T cells after TB Ag stimulation were observed between LTBI and TB groups. However, the BCG results again contrasted with those of TB Ag stimulation, with significantly (*q* = 0.001) higher numbers (Figure 6D) and frequencies (Supplementary Figure 3*B*) of circulating BCG-specific IFNγ ^+^ CD4^+^ and CD8^+^ T cells in LTBI donors.

Given that the higher levels of secreted IFNγ protein in patients with TB in the TB Ag stimulation condition were not due to T-cell differences, we next examined the transcriptional IFNγ response using nanostring assays on the stimulated whole blood cell pellet. This showed a significant difference (*q* = 0.02) between the 2 groups (Figure 6E) but to a lesser degree than the protein response, suggesting either differential kinetics or post-transcriptional regulatory mechanisms. For BCG, the transcriptional response (Figure 6F) mirrored the protein and cellular data, supporting the conclusion that patients with TB have lower numbers of circulating BCG-specific T cells, resulting in a reduced IFNγ response.

To further investigate why some patients with LTBI and TB did not secrete detectable levels of IFNγ in the TruC system, we developed and applied a Simoa digital ELISA with a limit of detection of 11 fg/mL. This technique identified secretion of IFNγ from all patients with TB and 17 of 24 LTBI donors (Figure 6G). Using this ultrasensitive readout, we examined the correlation between RNA transcription and protein secretion. The LTBI donors showed a strong correlation (*R*s = 0.78), while for patients with TB it was significant but weaker (*R*s = 0.57) (Figure 6H), again suggesting possible altered post-transcriptional regulation. In contrast, the RNA–protein correlation for both groups after BCG stimulation was strong (*R*s > 0.76) (Supplementary Figure 3C).

Finally, we examined how this ultrasensitive digital ELISA readout applied to TruC stimulation would classify patients with LTBI and TB. Utilizing the QFT equivalent cutoff of 14 pg/mL, we created 4 groups: LTBI IFNγ ^low^, LTBI IFNγ ^high^, TB IFNγ ^high^, and TB IFNγ ^low^ (Figure 6H). To test the potential biological relevance of this new classification, we performed unsupervised hierarchical clustering analysis based on the 50 most differential genes after TB Ag stimulation between LTBI and TB. This analysis showed that 4 of 5 LTBI IFNγ ^high^ individuals clustered with the active TB group when stimulated with TB Ag (Figure 6I) but not after BCG stimulation (Supplementary Figure 3D). Therefore, this combined approach of standardized whole-blood stimulation and digital ELISA may allow identification of additional stages within the spectrum of *Mtb* infection and disease, which is now recognized to include incipient and subclinical TB [17].

We conclude that the use of TruC may provide considerable advantages if further developed as a method for immunomonitoring in TB clinical studies and patient-management strategies.

## DISCUSSION

Blood-based immunomonitoring is increasingly used in clinical studies due to the ease of sampling and the possibility of longitudinal measurements during medical interventions. Tuberculosis is a relevant example of how such an approach can be applied to monitor functional immune responses in clinical applications, and this approach has been extended to cytomegalovirus infection and transplantation settings [18]. However, the use of QFT blood-based tests in TB-endemic countries has been limited by their poor ability to discriminate active TB disease from asymptomatic *Mtb* infection. Such stratification is required for proposed TB-control strategies that focus on preventive treatment to reduce risk for disease progression, thus diminishing the chance for *Mtb* transmission. This may potentially be achieved by TruC stimulation with heparin-binding hemagglutinin adhesin (HBHA), a mycobacterial antigen that has been shown to induce IFNγ preferentially in LTBI donors [19].

We demonstrated here a clear advantage of utilizing an alternative immunomonitoring tool, TruC, for the analysis of induced immune responses in TB disease. TruC showed significant differential IFNγ responses in patients with active disease and controls with LTBI, differences that have not been achieved using the QFT test [15]. Stimulation with BCG yielded a unique signature, with higher expression of multiple cytokines in LTBI as compared with active disease. This was at least partly explained by a reduced number of circulating BCG-specific T cells as revealed by flow cytometry. Combining the *Mtb* Ag and BCG-induced responses improved classification of active versus LTBI individuals significantly. The use of TruC also revealed differential induction of other cytokines, representing both innate and adaptive immune responses. Importantly, we show that such immune response differences may be obscured in QFT by cytokines that are activated nonspecifically, most likely from myeloid cells in the absence of liquid media.

The high concentrations of multiple cytokines in the control QFT tube were a striking observation. The elevated nonspecific immune responses were reminiscent of our previously reported nonspecific activation of myeloid cells [12]. To minimize such issues in clinical applications of QFT, decision making is restricted to IFNγ responses, with the nonstimulated control being subtracted from the *Mtb* Ag stimulation [5]. The manufacturer’s instructions for QFT-TB Gold and QFT-Plus permit up to 8 IU/mL of IFNγ within the unstimulated control before assigning the test as indeterminate, which is a high level of “background” response. While this delivers meaningful information about antigen-specific adaptive responses, our study illustrates the high value of reducing the overall background biological noise due to the method of stimulation [20], as it revealed previously unappreciated differences in antigen-specific signals that discriminate active TB and asymptomatic infection. We compared our results with published studies that utilized QFT stimulations and Luminex technology [21, 22, 23]. The ranges reported and those observed in our study were of a similar magnitude (eg, for IL-1RA, CXCL10^22^, IL-1α, IL-1β, IL-2, IL-6, CCL2, CCL3, TNFα [24]). Notable differences were observed in the levels of IL-8 and CCL4, which were an order of magnitude lower than those observed in our study. This may be explained by differences in antibodies used or the populations studied [25].

While an obvious caveat of our study are the relatively small sample sizes, we highlight that the high effect size seen between persons with LTBI and TB was great enough to observe a statistically significant difference, which was replicated in an independent cohort. The strong effect size also highlights the importance of assay standardization: lower variability obtained from more robust sampling can facilitate the powering of scientific questions, especially those with smaller study sizes. Additional studies are planned to identify clinical questions that would benefit from TruC-based immunomonitoring. Given recent advances in ex vivo blood transcriptomic signatures for diagnosing subclinical or active TB disease [26, 27], the requirement for an incubation step may represent a barrier to near-patient testing. Despite the stated limitations, we believe that there is sufficient justification for testing TruC as next-generation immunomonitoring tools in TB clinical studies.

In summary, given the numerous challenges still present in the TB field and the critical need for better tools, novel robust and adaptable immunomonitoring tools may support ongoing efforts to combat TB worldwide.

## Supplementary Data

Supplementary materials are available at *Clinical Infectious Diseases* online. Consisting of data provided by the authors to benefit the reader, the posted materials are not copyedited and are the sole responsibility of the authors, so questions or comments should be addressed to the corresponding author.

ciaa1562_suppl_Supplementary_Figure_S1

ciaa1562_suppl_Supplementary_Figure_S2

ciaa1562_suppl_Supplementary_Figure_S3

ciaa1562_suppl_Supplementary_Material

## Notes

***The Milieu Intérieur Consortium***. The Milieu Intérieur Consortium is composed of the following team leaders: Laurent Abel (Hôpital Necker), Andres Alcover, Hugues Aschard, Kalla Astrom (Lund University), Philippe Bousso, Pierre Bruhns, Ana Cumano, Caroline Demangel, Ludovic Deriano, James Di Santo, Françoise Dromer, Gérard Eberl, Jost Enninga, Jacques Fellay (L’École polytechnique fédérale de Lausanne [EPFL], Lausanne) Odile Gelpi, Ivo Gomperts-Boneca, Milena Hasan, Serge Hercberg (Université Paris 13), Olivier Lantz (Institut Curie), Claude Leclerc, Hugo Mouquet, Etienne Patin, Sandra Pellegrini, Stanislas Pol (Hôpital Côchin), Antonio Rausell (INSERM UMR 1163–Institut Imagine), Lars Rogge, Anavaj Sakuntabhai, Olivier Schwartz, Benno Schwikowski, Spencer Shorte, Vassili Soumelis (Institut Curie), Frédéric Tangy, Eric Tartour (Hôpital Européen George Pompidou), Antoine Toubert (Hôpital Saint-Louis), Mathilde Touvier (Université Paris 13), Marie-Noëlle Ungeheuer, Matthew L. Albert (Insitro; coordinator of the Milieu Intérieur Consortium), Darragh Duffy (coordinator of the Milieu Intérieur Consortium), Lluis Quintana-Murci (coordinator of the Milieu Intérieur Consortium). Additional information can be found at: http://www.milieuinterieur.fr/en.

Unless otherwise indicated, partners are located at Institut Pasteur, Paris.

***Acknowledgments.*** The authors thank the UTechS CB and Icareb platforms of the Center for Translational Research, Institut Pasteur. We are grateful to the study participants and the SATVI clinical and laboratory teams.

***Financial support.*** This work was supported by the Bill and Melinda Gates Foundation (grant number OPP1114368). Additional support was provided by the French Government’s Investissement d’Avenir Program, Laboratoire d’Excellence “Milieu Intérieur” grant ANR-10-LABX-69-01. Clinical collection of TB patient samples included in the replication cohort was supported by the Bill and Melinda Gates Foundation (OPP1155128), the European and Developing Countries Clinical Trials Partnership (96425), and the Intramural Research Program of the National Institute of Allergy and Infectious Diseases and the National Institutes of Health (1U01AI115619-01). E. N. is an International Society for Advancement of Cytometry (ISAC) Marylou Ingram Scholar and A. L. was supported by the Fondation Recherche Médicale (SPF20170938617).

***Potential conflicts of interest.*** M. L. A. is an employee of Insitro. D. D. reports other support from Myriad Rules Based Medicine, outside the submitted work. G. W. reports 2 issued patents on TB diagnostic markers issued (PCT/IB2013/054377; host markers in whole-blood culture supernatants for diagnosis for active TB; and PCT/IB2013/054377 [USA]; host markers in whole-blood culture supernatants); a patent on a method for diagnosing TB pending (AP/P/2016/009427 [ARIPO], 201580023042.X [China], 15755681.2 [Europe], 201617030869 [India], F/P/2016/258 [Nigeria], 2016/06324 [South Africa]); and a patent on serum host biomarkers for TB disease pending (PCT/IB2017/052142, due for national filing). All other authors report no potential conflicts. All authors have submitted the ICMJE Form for Disclosure of Potential Conflicts of Interest. Conflicts that the editors consider relevant to the content of the manuscript have been disclosed.
