# Supplementary figures and images for: Immune Profiling Enables Stratification of Patients With Active Tuberculosis Disease or Mycobacterium tuberculosis Infection
Source: Clin Infect Dis. 2020 Oct 16;73(9):e3398–408. doi: 10.1093/cid/ciaa1562 (PMC8563210; doi:10.1093/cid/ciaa1562)

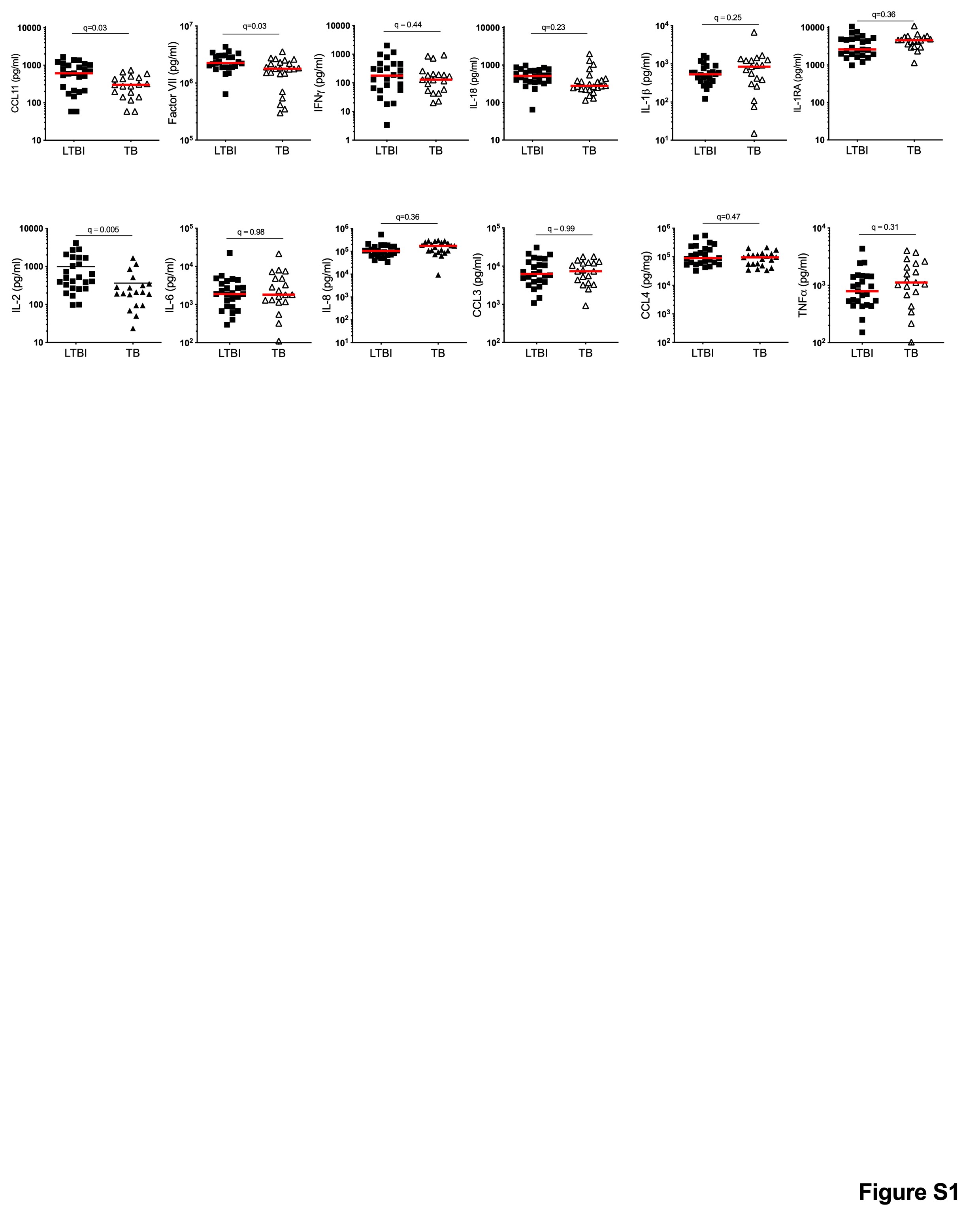

Supplement: ciaa1562_suppl_Supplementary_Figure_S1 [file ciaa1562_suppl_supplementary_figure_s1.jpeg]

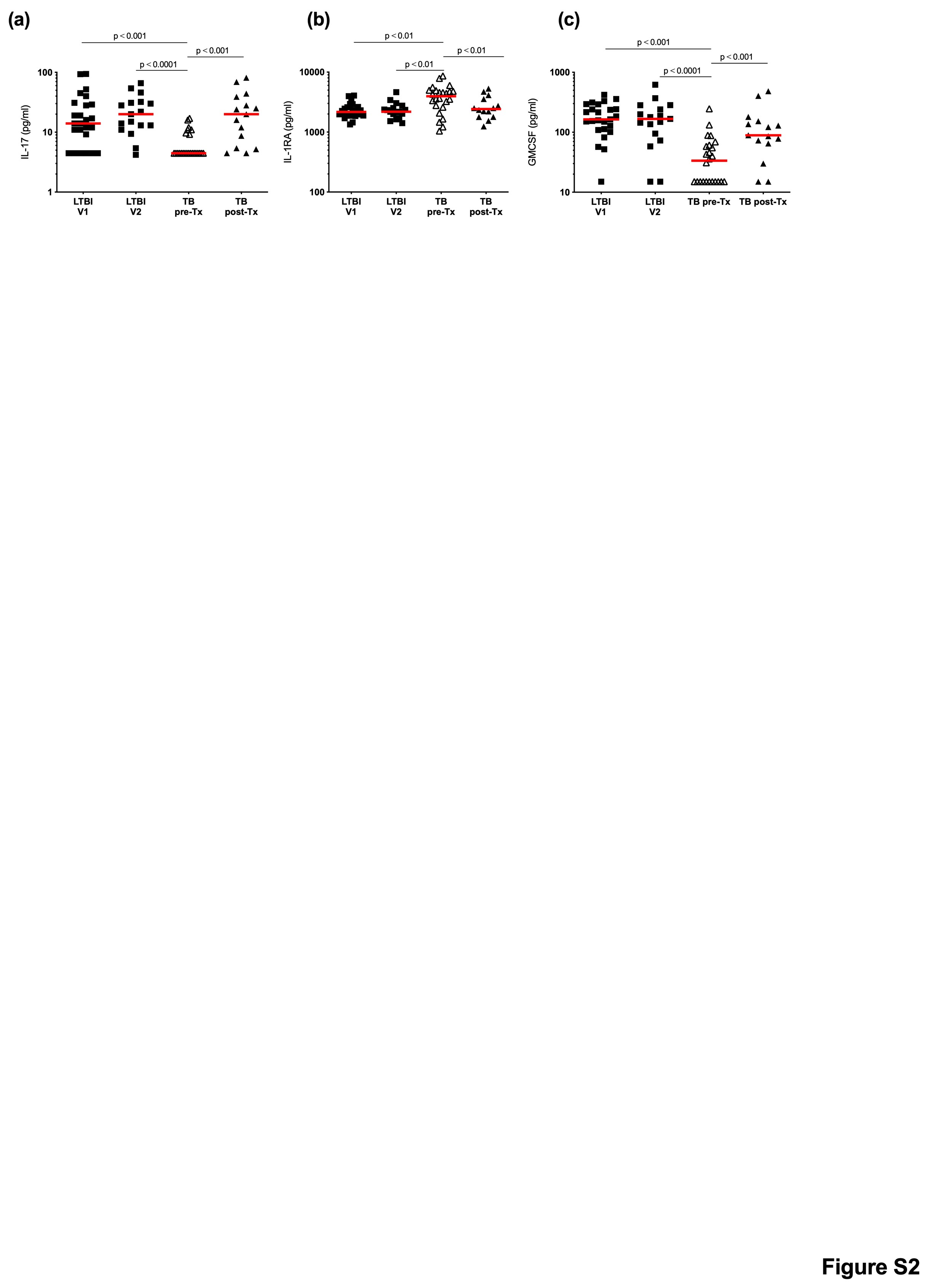

Supplement: ciaa1562_suppl_Supplementary_Figure_S2 [file ciaa1562_suppl_supplementary_figure_s2.jpeg]

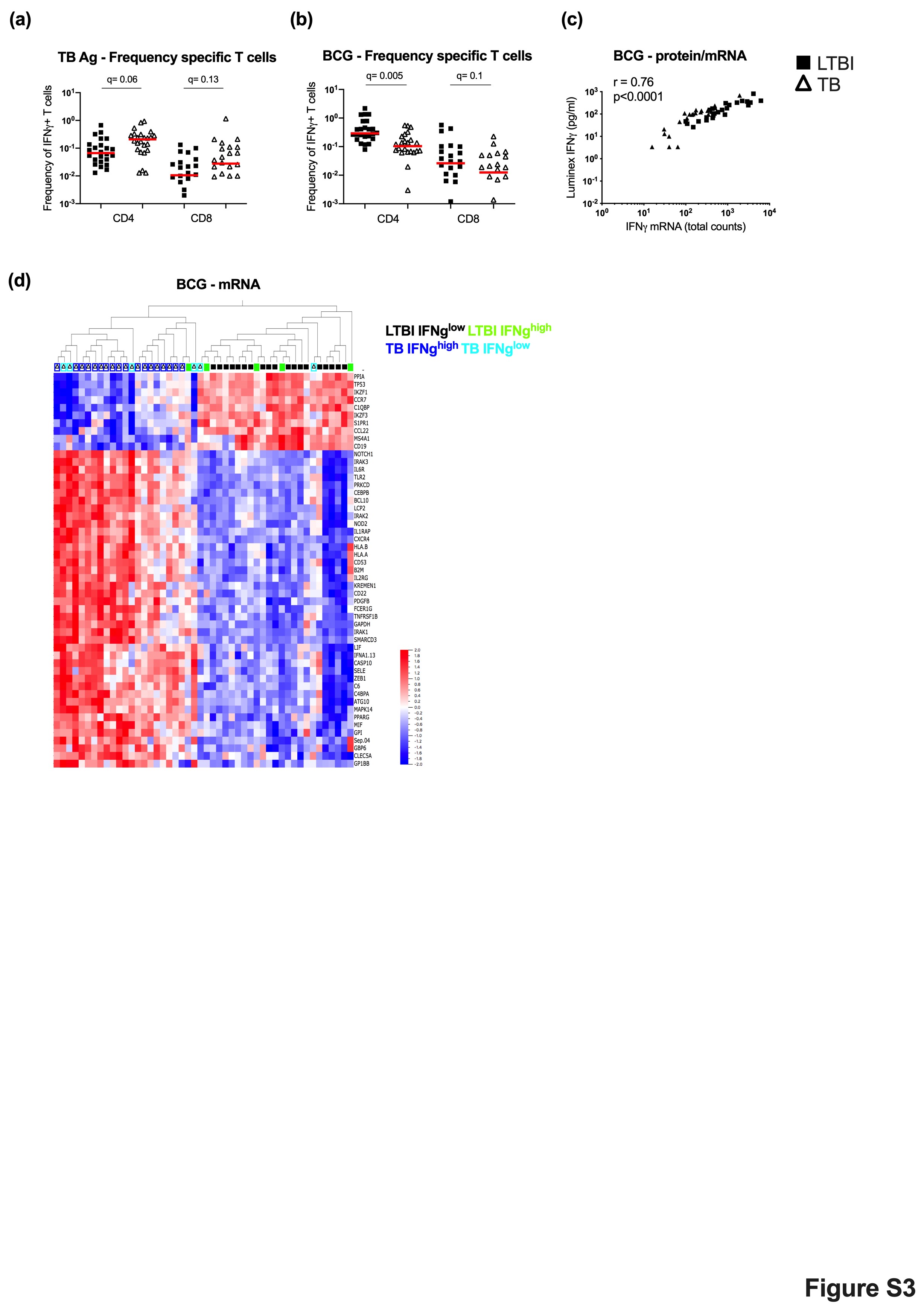

Supplement: ciaa1562_suppl_Supplementary_Figure_S3 [file ciaa1562_suppl_supplementary_figure_s3.jpeg]
