# Supplementary material for: Immune Profiling Enables Stratification of Patients With Active Tuberculosis Disease or Mycobacterium tuberculosis Infection
Source: Clin Infect Dis. 2020 Oct 16;73(9):e3398–408. doi: 10.1093/cid/ciaa1562 (PMC8563210; doi:10.1093/cid/ciaa1562)
Supplement: ciaa1562_suppl_Supplementary_Material [file ciaa1562_suppl_supplementary_material.docx]

Supplemental Methods

*Participant groups*

25 healthy adults with asymptomatic, latent *Mtb* infection (LTBI), defined by a positive QuantiFERON-TB Gold In-Tube (QFT+) assay (Qiagen, Germany), and 25 HIV-negative adults with TB disease (TB), defined by a positive sputum XpertMTB/RIF test (Cepheid, United States) were identified and recruited at the South African Tuberculosis Vaccine Initiative (SATVI), Worcester, South Africa^11^. The LTBI control group was age, sex and ethnicity matched as closely as possible to the active TB disease patient group. Blood was collected prior to treatment initiation (V1) in active TB cases and again 12-18 months later, after successful completion of treatment and having been declared clinically cured (V2, n=18). For the LTBI controls, blood was also collected at a second time-point, 12-18 months after the initial visit (V2, n=19). TB patient and LTBI control characteristics are described in Table 1. For the replication cohort: TB patients (n=51) enrolled in the PREDICT trial (Clinicaltrials.gov NCT02821832) at SATVI, were co-enrolled into this biomarker study. LTBI controls (n=9), recruited at SATVI and healthy donors (n=10), recruited in Paris, France, were also included. Stimulation was performed with Null and TB antigen (ESAT-6, CFP-10, TB7.7) TruC tubes (see below), and analysis was performed in a blinded fashion. The TB clinical studies, protocols and informed consent forms were approved by the Human Research Ethics Committee of the University of Cape Town (ref: 234/2015). Healthy donor blood from a French population in a non-endemic TB setting was obtained from the CoSImmGEn cohort of the Investigation Clinique et Accès aux Ressources Biologiques (ICAReB) platform, Centre de Recherche Translationnelle, Institut Pasteur, Paris, France. Written informed consent was obtained from all study participants.

*Whole blood stimulations*

1mL of whole blood was collected directly in QFT Gold tubes (Nil, TB Antigens, Mitogen) (Qiagen) according to manufacturers’ instructions. Whole blood was also collected in Sodium Heparin tubes and 1mL transferred into TruC tubes (total volume 3mL). Unstimulated control tubes from both stimulation systems are referred to as Null throughout to avoid confusion. QFT and TruC tubes were processed within 30 minutes of blood draw, inserted into a dry block incubator, and maintained at 37°C (±1°C) room air for 22 hrs (+/- 15 minutes) as previously described^7^. At the end of the incubation period, QFT tubes were centrifuged and TruC tubes were opened and a valve was inserted to separate the sedimented cells from the supernatant, stopping the stimulation reaction. Separate supernatant aliquots were prepared for ELISA and Luminex testing and frozen at -80°C until analysis. Cell pellets were stabilized in Trizol LS for transcriptional analysis as previously described^8^.

*Multi-analyte protein profiling*

Samples were measured according to CLIA guidelines (set forth by the USA Clinical and Laboratory Standards Institute). The 32 measured analytes were organized on 3 multiplex arrays, and a single batch of reagents was used for testing all samples per timepoint. The least detectable dose (LDD) for each assay was derived by averaging the values obtained from 200 runs with the matrix diluent and adding 3 standard deviations to the mean. The lower limit of quantification (LLOQ) was determined based on the standard curve for each assay and is the lowest concentration of an analyte in a sample that can be reliably detected and at which the total error meets CLIA requirements for laboratory accuracy. The lower assay limit (LAL) is the lowest value read out after application of the standard curve and use of curve-fitting algorithms. In most instances, the LAL is less than the LDD and the LLOQ. To enable a direct comparison between both stimulation systems protein concentrations were calculated to pg/ml of whole blood, which integrated the original dilution factors.

*Simoa digital ELISA*

To detect low concentrations of IFNγ a Homebrew Simoa ELISA was developed as previously described^12^. The capture antibody (MD-1, BioLegend) was used at 0.3 mg/ml and the detector antibody (25718, R&D systems) was conjugated at 40x biotin:antibody ratio and used at 0.3 μg/ml. Streptavidin-β-galactosidase was used at a final concentration of 150 pM. Limit of detection (LoD) was defined as blank + 3SD and was 11 fg/ml. The assay was run on a 2-step configuration on the Simoa HD-1 Analyzer (Quanterix, US) and cytokine values were expressed in pg/ml.

*Nanostring transcriptional analysis*

Nanostring gene expression analysis was performed following extraction of RNA from Trizol stabilized TruC cell pellets as previously described and detailed in the supplemental methods ^8^. RNA integrity was assessed with Agilent RNA kits for the 2100 Bioanalyzer System and the NanoString nCounter system was used for the digital counting of transcripts. RNA was quantified using the Qubit RNA HS Assay Kit (Thermo Fischer Scientific) and 100 ng of total RNA hybridized with the Human Immunology v2 (plus 30 additional TB relevant genes listed in Table S1) Gene Expression CodeSet according to the manufacturer’s instructions. Samples were normalized following background subtraction of the negative control probes, using positive control probes and Housekeeping genes (SDHA, HPRT1, POLR2A, RPL19, G6PD, TBP) selected by the GNorm method as previously described^9^ using the nSolver^TM^ analysis software (NanoString technologies). Quality control for our data involved checking the following metrics: fields of view counted (flag if < 0.75), binding density (flag if not in the 0.05-2.75 range), linearity of positive controls (flag if R2 < 0.9) and limit of detection for positive controls (flag if 0.5 fM positive control < 2 SDs above the mean of the negative controls).

*Statistical analysis*

Non-parametric T tests (Mann Whitney) were performed for two group comparisons with Qlucore Omics Explorer, v.3.5 (Qlucore) or GraphPAD Prism (version 8). For comparisons between pre and post treatment paired *t* tests (Wilcoxon) were performed with GraphPAD Prism. Multiple testing correction was performed and false discovery rate (FDR)-adjusted p values (q values) are reported. For the analysis of donors in a non-endemic setting, Friedman test with Dunn’s multiple comparisons test were applied for comparing multiple conditions (n=10). Dot plot graphs were compiled with GraphPad Prism and heat maps were generated using Qlucore v.3.5. Receiver operating characteristic (ROC) curves were calculated and compared with R Studio v.3.3.1 pROC package and results drawn with graphical package ggplot2 v.2.1.0. Sample size calculation was performed and 25 persons per group was chosen to have sufficient power to detect differences due to TB disease in the initial discovery cohort. Calculations were based on previously published induced immune responses in *Mtb* infected vs. healthy controls^5^.

**Table S1.** Tuberculosis-related genes added to the Nanostring human immunology v2 panel.

**Table S2**. Q values from Mann-Whitney testing between TB and LTBI groups following *Mtb* Ag stimulation with TruC or QFT tubes, and median values with inter-quartile (IQ) ranges in pg/mL.

**Table S3**. Median values with inter-quartile (IQ) ranges in pg/mL of LTBI and TB groups in the Null condition of TruC tubes.

**Table S4**. Median values with inter-quartile (IQ) ranges in pg/mL of LTBI and TB groups in the Null condition of QFT tubes.

**Table S5**. Q values from Mann-Whitney testing between TB and LTBI groups following BCG stimulation with TruC tubes, and median values with inter-quartile (IQ) ranges in pg/mL.

| **Gene name** |
| --- |
| ACTA2 |
| ALDH1A1 |
| ANKRD22 |
| APOL1 |
| APOL6 |
| BATF2 |
| KLF2 |
| CALML4 |
| CASP4 |
| CREB5 |
| CYB561 |
| DEFA1 |
| DUSP3 |
| ETV7 |
| GAS6 |
| GBP2 |
| GBP4 |
| GBP6 |
| HPSE |
| KCNJ15 |
| KREMEN1 |
| LACTB |
| LHFPL2 |
| LOC389386 |
| FER1L3 |
| SCARF1 |
| SEPT4 |
| SMARCD3 |
| TRAFD1 |
| VAMP5 |

Table S1. Tuberculosis-related genes added to the Nanostring human immunology v2 panel.

| **TruC** | | | | | | | |
| --- | --- | --- | --- | --- | --- | --- | --- |
| **Protein** | **LTBI v TB**  **(q values)** | **LTBI** | | | **TB** | | |
|  |  | **IQR1** | **Median** | **IQR2** | **IQR1** | **Median** | **IQR2** |
| CCL11 | 0.004 | 58.5 | 231 | 344 | 58.5 | 58.5 | 188 |
| Factor VII | 0.001 | 261000 | 298000 | 319000 | 195250 | 230000 | 268000 |
| IFNγ | 0.0001 | 3.4 | 3.4 | 7 | 28.25 | 95.5 | 159.75 |
| IL-18 | 2.9 x10^-6^ | 105 | 121 | 161 | 228.25 | 279 | 404.75 |
| IL-1β | 0.0007 | 4.25 | 4.25 | 4.25 | 9.75 | 18.5 | 43.25 |
| IL-1RA | 7.8 x10^-6^ | 73 | 98 | 146 | 571.5 | 1410 | 2067.5 |
| IL-2 | 0.001 | 23.5 | 23.5 | 23.5 | 42.625 | 95.5 | 183.25 |
| IL-6 | 0.0001 | 1.7 | 4.1 | 9.8 | 23.5 | 39.5 | 71.5 |
| IL-8 | 2.9 x10^-6^ | 185 | 359 | 1230 | 5677.5 | 14500 | 21525 |
| CCL3 | 0.001 | 37 | 75 | 154 | 387.75 | 768 | 1857.5 |
| CCL4 | 0.001 | 1290 | 2520 | 4020 | 16887 | 32050 | 58525 |
| TNFα | 0.001 | 9 | 19 | 35 | 49.75 | 135.5 | 288.75 |

| **QFT** | | | | | | | |
| --- | --- | --- | --- | --- | --- | --- | --- |
| **Protein** | **LTBI v TB**  **(q values)** | **LTBI** | | | **TB** | | |
|  |  | **IQR1** | **Median** | **IQR2** | **IQR1** | **Median** | **IQR2** |
| CCL11 | 0.03 | 212 | 610 | 1020 | 166 | 302 | 452.5 |
| Factor VII | 0.03 | 1890000 | 2250000 | 3010000 | 1460000 | 1780000 | 2250000 |
| IFNγ | 0.44 | 65 | 169 | 459 | 76.5 | 168 | 232.5 |
| IL-18 | 0.23 | 361 | 511 | 710 | 507 | 1080 | 1450 |
| IL-1β | 0.25 | 355 | 534 | 763 | 342.5 | 844 | 1250 |
| IL-1RA | 0.98 | 1920 | 2600 | 4850 | 3045 | 4580 | 5490 |
| IL-2 | 0.005 | 266 | 499 | 1670 | 185 | 211 | 334.5 |
| IL-6 | 0.98 | 898 | 1880 | 3520 | 1235 | 1810 | 4840 |
| IL-8 | 0.36 | 67300 | 103000 | 157000 | 105000 | 173000 | 240000 |
| CCL3 | 0.99 | 3920 | 6270 | 12600 | 4005 | 7360 | 12200 |
| CCL4 | 0.47 | 60100 | 89600 | 188000 | 55600 | 95300 | 116000 |
| TNFα | 0.31 | 530 | 790 | 1430 | 724 | 1120 | 2240 |

Table S2. Q values from Mann-Whitney testing between TB and LTBI groups following *Mtb* Ag stimulation with TruC or QFT tubes, and median values with inter-quartile (IQ) ranges in pg/mL.

| **TruC** | | | | | | |
| --- | --- | --- | --- | --- | --- | --- |
| **Protein** | **LTBI** | | | **TB** | | |
|  | **IQR1** | **Median** | **IQR2** | **IQR1** | **Median** | **IQR2** |
| BDNF | 10000 | 13000 | 16000 | 9275 | 11000 | 13250 |
| CCL11 | 58.5 | 246 | 399 | 58.5 | 129 | 213.75 |
| Factor VII | 237000 | 292000 | 318000 | 183750 | 231500 | 246500 |
| ICAM | 57000 | 71000 | 80000 | 71000 | 82500 | 166250 |
| IFN*γ* | 3.4 | 3.4 | 3.4 | 3.4 | 3.4 | 3.4 |
| IL-10 | 2.6 | 2.6 | 2.6 | 2.6 | 2.6 | 2.6 |
| IL-12p40 | 225 | 225 | 490 | 515 | 805 | 1325 |
| IL-15 | 600 | 600 | 600 | 750 | 750 | 750 |
| IL-18 | 100 | 127 | 137 | 385.5 | 566.5 | 810 |
| IL-1β | 4.25 | 4.25 | 4.25 | 213 | 336 | 515.75 |
| IL-1RA | 64 | 78 | 114 | 900.75 | 1250 | 1945 |
| IL-2 | 23.5 | 23.5 | 23.5 | 32.5 | 32.5 | 32.5 |
| IL-23 | 1600 | 1600 | 1600 | 1012.5 | 2300 | 2650 |
| IL-6 | 1.7 | 1.7 | 1.7 | 205.25 | 872.5 | 2540 |
| IL-8 | 34 | 100 | 154 | 43825 | 77150 | 92300 |
| CCL2 | 214 | 389 | 569 | 5035 | 21550 | 32550 |
| CCL3 | 12.5 | 32 | 40 | 2120 | 4655 | 8400 |
| CCL4 | 564 | 689 | 1140 | 34100 | 53300 | 106600 |
| MMP3 | 6100 | 8300 | 13000 | 33500 | 40500 | 65500 |
| MMP9 | 16500 | 16500 | 16500 | 22500 | 22500 | 22500 |
| SCF | 111 | 111 | 111 | 127 | 280 | 445 |
| TNFα | 9 | 9 | 9 | 306.25 | 528.5 | 925.75 |
| VEGF | 294 | 328 | 478 | 312.5 | 689.5 | 954 |

Table S3. Median values with inter-quartile (IQ) ranges in pg/mL of LTBI and TB groups in the Null condition of TruC tubes.

| **QFT** | | | | | | |
| --- | --- | --- | --- | --- | --- | --- |
| **Protein** | **LTBI** | | | **TB** | | |
|  | **IQR1** | **Median** | **IQR2** | **IQR1** | **Median** | **IQR2** |
| BDNF | 13000 | 21000 | 25000 | 14500 | 17500 | 27500 |
| CCL11 | 267 | 827 | 1100 | 150 | 366 | 597.5 |
| Factor VII | 2120000 | 2430000 | 3020000 | 1470000 | 1760000 | 2330000 |
| ICAM | 243000 | 532000 | 630000 | 610000 | 733000 | 1092500 |
| IFN*γ* | 3.4 | 3.4 | 7 | 3.4 | 7.6 | 14.25 |
| IL-10 | 12 | 16 | 33 | 14.5 | 19 | 28.5 |
| IL-12p40 | 880 | 1100 | 1300 | 660 | 830 | 875 |
| IL-15 | 1300 | 1500 | 1600 | 600 | 1200 | 1300 |
| IL-18 | 390 | 524 | 696 | 518.25 | 869 | 1562.5 |
| IL-1β | 147 | 505 | 664 | 147 | 333 | 882.5 |
| IL-1RA | 647 | 987 | 1250 | 967 | 1350 | 1730 |
| IL-2 | 23.5 | 23.5 | 23.5 | 23.5 | 23.5 | 23.5 |
| IL-23 | 4000 | 5000 | 5600 | 1600 | 3700 | 4400 |
| IL-6 | 768 | 2120 | 2820 | 251 | 1420 | 3890 |
| IL-8 | 37700 | 58100 | 77900 | 50100 | 92800 | 171000 |
| CCL2 | 4540 | 7930 | 14300 | 7212.5 | 14300 | 30075 |
| CCL3 | 3910 | 4860 | 7520 | 1075 | 4415 | 11800 |
| CCL4 | 33500 | 40900 | 59000 | 22050 | 36200 | 61550 |
| MMP3 | 39000 | 58000 | 90000 | 36500 | 43500 | 61500 |
| MMP9 | 16500 | 48000 | 52000 | 16500 | 41000 | 69000 |
| SCF | 430 | 569 | 780 | 404.5 | 555 | 659 |
| TNFα | 456 | 805 | 1290 | 198.25 | 674 | 1550 |
| VEGF | 564 | 796 | 901 | 738 | 1330 | 1595 |

Table S4. Median values with inter-quartile (IQ) ranges in pg/mL of LTBI and TB groups in the Null condition of QFT tubes.

| **TruC - BCG** | | | | | | | |
| --- | --- | --- | --- | --- | --- | --- | --- |
| **Protein** | **LTBI v TB**  **(q values)** | **LTBI** | | | **TB** | | |
|  |  | **IQR1** | **Median** | **IQR2** | **IQR1** | **Median** | **IQR2** |
| GMCSF | 2.1x10^-5^ | 113 | 164 | 242 | 15 | 33.5 | 58.75 |
| TNFα | 0.004 | 5300 | 6650 | 11200 | 1742.5 | 2850 | 6472.5 |
| IL-1β | 0.0008 | 2520 | 4420 | 5670 | 855 | 1590 | 2362.5 |
| IL-1α | 0.0006 | 46 | 84 | 110 | 11 | 25.5 | 38 |
| IL-12p40 | 0.003 | 1100 | 1300 | 1500 | 485 | 770 | 1125 |
| CCL2 | 0.003 | 26500 | 38800 | 65500 | 10092.5 | 16900 | 29500 |
| IL-3 | 0.003 | 9.7 | 12 | 13 | 6.675 | 8.6 | 10 |
| IL-17 | 0.0008 | 9.2 | 14 | 29 | 4.45 | 4.45 | 9.325 |
| IL-18 | 4.5x10^-5^ | 171 | 189 | 226 | 268 | 353.5 | 498.75 |
| IL-1RA | 0.003 | 1910 | 2170 | 2620 | 2727.5 | 3960 | 4830 |

Table S5. Q values from Mann-Whitney testing between TB and LTBI groups following BCG stimulation with TruC tubes, and median values with inter-quartile (IQ) ranges in pg/mL.
